# Community health nurses' concerns about infant regulatory problems are predictive of mental disorders diagnosed at hospital: a prospective cohort study

**DOI:** 10.3389/frcha.2023.1330277

**Published:** 2024-01-15

**Authors:** Sofie Weber Pant, Bjørn Evald Holstein, Janni Ammitzbøll, Anne Mette Skovgaard, Trine Pagh Pedersen

**Affiliations:** National Institute of Public Health, University of Southern Denmark, Copenhagen, Denmark

**Keywords:** children, community health nurses, incessant crying, eating problems, infants, mental disorders, sleeping problems, regulatory problems

## Abstract

**Introduction:**

Regulatory problems of eating, sleeping, and crying in infancy may index mental health vulnerability in older ages, and knowledge is needed to inform strategies to break the developmental trajectories of dysregulation in early childhood. In this study, we examined the prospective associations between infant regulatory problems at the age of 8–10 months identified by community health nurses (CHN) and mental disorders diagnosed in hospital settings in children aged 1–8 years.

**Methods:**

From a cohort of all newborn children in 15 municipalities in the Capital Region of Copenhagen (*N* = 43,922) we included all children who were examined by CHNs at the scheduled home visit at the age of 8–10 months (*N* = 36,338). Outcome measures were ICD-10 mental disorders diagnosed at public hospitals and reported to the National Patient Register. Logistic regression included data on child and family covariables obtained from population registers.

**Results:**

The CHNs reported concerns regarding sleep in 7.7% of the study population, feeding and eating in 19.1%, combined sleeping and eating problems in 3.6%, and incessant crying in 0.7%. A total of 1,439 children (4% of the study population) were diagnosed in hospital settings with an ICD-10 mental disorder between the ages of 11 months and 8 years. Analyses adjusted for a range of perinatal and family adversities showed an increased risk of any neurodevelopmental disorder among children with CHN concerns of feeding and eating (odds ratio (OR) 1.36 (95% confidence interval (CI) 1.14–1.63)) and co-occurrent problems of feeding and eating and sleep (OR 1.60 (95% CI 1.14–2.26)). For autism-spectrum disorders, an increased risk was seen among children with co-occurrent problems of both feeding and eating and sleep (OR 1.73 (95% CI 1.07–2.79)). Concern about feeding and eating was also associated with an increased risk of behavioral and emotional disorders (OR 1.27 (95% CI 1.03–1.56)). Concern about incessant crying at the age of 8–10 months was not associated with a diagnosed mental disorder, but findings may reflect low statistical power due to low frequency of concern.

**Discussion:**

CHN concerns mirror a group of developmentally vulnerable children. Further research is needed to explore the possibilities of preventive intervention within the general child health surveillance to address the developmental psychopathology of dysregulation in early ages.

## 1 Introduction

Persistent problems of feeding, sleeping, and incessant crying in infancy are often referred to as infant regulatory problems (RP) (1–3). RPs represent the extreme on a continuum of problem severity, ranging from normal variations to problems that exceed deviations in the normal development, overall influencing the wellbeing of the child (1–3). Infants’ regulation of sleep, feeding and eating, and emotional reactions matures during the early postnatal months, highly influenced by innate vulnerabilities and the external regulatory support provided by the parents. The first 4 months of life are a period of adaptation and maturation in which transitory problems of regulation is frequent (3). The current conceptualization of RPs includes problems that are pervasive and persistent over time, and exceeding the variations being considered within the normal developmental range (4). Still, the concept and definition of RPs in infancy is not fully validated (5, 6). Different criteria and questions are used across studies to define when the problems are outside the normal range (3, 5).

According to the available literature, RPs may affect one-fifth of all infants (1, 2, 4). There is an increased risk seen in children born with perinatal adversities, as well as children from families of psychosocial adversities, and in particular maternal mental health problems and parent–child relational problems (2). Longitudinal data have found that RPs in infancy are associated with a wide range of mental health problems in childhood and adolescence (1, 3–21). The frequency and possible developmental impact of RPs point to public health approaches to the early detection and interventions addressing infants with RP. This is to reduce the developmental risk of mental health problems in childhood and beyond, as well as the long-term consequences of such problems (22–24).

A meta-analysis from 2011 that included clinical/high-risk as well as community-based studies found an overall increased risk of behavioral problems, externalizing, and attention-deficit/hyperactivity disorder (ADHD) later in childhood among children with RPs in infancy (1). A meta-analysis from 2023 (6) found a cumulative risk of behavioral problems of 23.3% for children with RPs compared to 6.7% for healthy controls. The outcome measures included externalizing behavior, internalizing behavior, and ADHD, most often assessed by parents' use of rating scales. The meta-analysis did not find any important subgroup differences or moderating effects among children with infancy RPs. The effect sizes were comparable regardless of the follow-up age. In addition, studies exploring the neurodevelopmental outcome of infancy RPs in clinical as well as population-based cohorts have found associations with autism spectrum disorders (ASD) and ADHD (3, 5, 7–14, 16–19, 21, 25).

The co-occurrence of two or more RPs has consistently been found associated with an increased cumulative risk of mental disorders (3, 4, 18). Moreover, co-occurrent or combined RPs have been suggested to be infancy markers of early dysregulation vulnerability tracking into the emotional, cognitive, and behavioral dysregulation of a range of mental disorders later in development (4, 18). Among the potential perinatal and psychosocial risk factors investigated, particularly preterm birth, maternal mental health problems and parent–child relationship problems have been found associated with increased developmental risk in infants with RPs (2, 14, 25–27). Altogether, the available evidence suggests that RPs in infancy may predict psychopathology across childhood and beyond (18). These suggestions point to the identification of RPs in infancy providing a window of opportunity to target intervention toward developmental cascades of dysregulation early in childhood through strategies to improve the parenting of vulnerable infants (2–4, 23).

Within the community child healthcare, a window of opportunity to target infancy RPs may be provided by health professionals working with infants and their families. In Denmark, the municipality child healthcare includes scheduled home visits from community health nurses (CHNs) to all families with a newborn child. These services are free of charge and attended by more than 95% of families with infants (20, 28). The CHNs are registered nurses with a 1.5-year formalized education, including the theoretical and clinical aspects of child assessments and communication with parents. In accordance with the recommendations from the National Health Authority, the CHNs offer at least five home visits during the first postnatal year. The main agenda of the home visits is the CHN’s evaluation of the child's physical and mental health, and a dialog with the parents about the developmental needs of the child, including action regarding further support or referrals if needed. In connection with the home visits, the CHN fills in a standardized record. The record is based on the CHN’s examination and observation of the child and on a conversation with the parents and is an overall conclusion regarding the child's development and wellbeing (20, 28, 29). If the child does not develop age-appropriately, the CHN records a concern for that area, including concern relating to sleep, eating, or crying. In the international literature, the CHN’s recordings are described as a concern, attention, or red flag, which indicate that something is different, wrong, odd, or troubling (30).

Although there are many studies that demonstrate an association between RP in infancy and mental health problems in childhood, there are still many unsolved issues. For instance, only few studies investigated the mental disorders according to defined diagnostic criteria, such as the criteria of the International Classification of Diseases, ICD-10 (5, 14). In addition, only few studies included a broad range of mental disorders as outcome measures (1, 4, 6). Further, the pattern of association between specific kinds of dysregulation and mental health problems varied across studies, and so did the effect sizes. Yet another unsolved issue relates to confounding due to well-established risk factors underscoring the need for comprehensive adjustment for confounders in studies of the prospective association between RPs in infancy and mental disorders in childhood.

The aim of this study was to examine the prospective association between CHN-observed concerns of RPs in infancy and child mental disorders diagnosed within hospital settings. The main hypothesis was that indicators of RPs identified in municipality settings during infancy would be predictive of mental disorders diagnosed later in childhood.

## 2 Methods

### 2.1 Study design and study population

The study is based on a prospective cohort of children followed from birth to their eighth birthday. The study included data on the standardized recordings from home visits from municipalities who are part of the Child Health Database collaboration. These recordings contain a range of information on family factors and child development, and they include recordings of the CHN's concerns about sleeping problems, eating problems, and incessant crying. Among the available data, we used information about RPs from the home visit when the child was aged 8–10 months, considering that problems within the first 6 months may be transitory and fading (2, 20). The records also included the child's unique person identification number making it possible to link data from the CHN’s recordings to data from Danish national registers (the National Birth Register, the Danish National Patient Register, and the Civil Registration System). For the present study, we applied the following criteria of inclusion criteria: (1) all newborns from 15 municipalities in the Copenhagen region in Denmark, representing a mixture of metropolitan, urban, and rural areas; (2) children born between 1 January 2002 and 31 December 2010, i.e., they had their eighth birthday before 1 January 2019; (3) having data about regulatory problems and other requested data from the CHN records; (4) no diagnosed mental disorder before the age of 11 months; and (5) having data about all applied covariates (confounder variables). The total number of children born in the 15 municipalities between 2002 and 2010 was 43,922. The eligible study population included the 36,338 children who complied with the inclusion criteria. Not all children had data on all three RPs explored (*N* = 9,712) and only for a few children had the CHN registered a concern for all three regulation problems (*n* = 11). Because of that, we did not conduct analyses with combined data on all three RPs. The analyses of sleeping problems used a subpopulation of 31,322 children, the analyses of eating problems used a subpopulation of 26,437 children, the analyses of combined sleeping and eating problems used a subpopulation of 25,960 children, and the analyses of incessant crying used a subpopulation of 13,987 children.

### 2.2 Measures

The *predictor variables* were regulatory problems at the age of 8–10 months derived from the CHNs' records. The CHN registered a concern in the child's record at the home visit at the age of 8–10 months if the CHN had concerns about RPs. The records are based on the overall conclusion from the parents' information and observations of the child during the home visit. To optimize the reliability of the recordings, the CHN used a manual of definitions. The CHN registered a concern regarding the following: (1) *sleep* if the child had not developed a stable sleep pattern, or if the child's sleep did not meet the child's need for sleep, summarized as a dichotomous variable, +/– concern regarding sleep; (2) *feeding and eating* in case of deviation from this definition: “The child gets a combination of breast milk/formula and solid food; there are no eating problems; the child drinks from a cup; the food meets the nutritional recommendations from the health authorities”, summarized as a dichotomous variable, +/– concern regarding feeding and eating, and (3) *incessant crying* if it is not possible to comfort the child when crying, summarized as a dichotomous variable, +/– concern regarding incessant crying. The term *concern* is a concept traditionally used by Danish CHNs to describe worrisome or risky conditions in the child or the caregiver environment (22), just like the concept's attention and red flag (30).

The main *outcome variable* was any mental disorder diagnosed in clinical settings at hospitals from a child aged 11 months to the child's eighth birthday. The mental disorders comprise all F-diagnoses (F00–F99) from the ICD-10 Classification of Mental and Behavioral Disorders: clinical descriptions and diagnostic guidelines (31). This included general developmental disorders (F70–F79), specific developmental disorders (F80–83), pervasive and other developmental disorders (F84–89), hyperkinetic disorders (F90), attention-deficit disorder without hyperactivity (F98.8), mood disorders, emotional and stress-related disorders (F30–F34, F38–F45, F48, F93), disorders of eating and sleep (F50–F51, F98.2), disorders of behavioral (F91–F92), and of social functioning (F94.1–F94.2, F94.8). Information on mental disorders in clinical settings was obtained from the Danish National Patient Register, which included all hospital contacts with a 100% coverage (32). Referrals and treatments at hospitals are free of charge in Denmark. The clinical diagnostic assessments included examinations in pediatric and psychiatric in- and outpatient and emergency settings completed by medical doctors in accordance with the diagnostic criteria of ICD-10 (33).

We summarized the outcome data into three dichotomous variables: at least one diagnosed mental disorder between the ages of 11 months and 8 years (yes, no), at least one diagnosed neurodevelopmental disorder (34), including intellectual disability, autism-spectrum disorders and disorders of hyperactivity and inattention (yes, no), and at least one diagnosed behavioral or emotional disorder (yes, no).

The analyses included a range of *covariates* from three sources. From the *National Birth Register*: gestational age (born in the 37th week or later vs. before); birth weight (<2,500, 2,500–3,999, and >3,999 g); congenital malformation (yes, no); mother's age at childbirth (<25 vs. ≥25); father's age at childbirth (<25 vs. ≥25); pregnancy complications (yes, no); cesarean section (yes, no); and Apgar score (9–10 vs. less). From the *Civil Registration System*: parents' education at childbirth (5 or more years of completed university education, other higher education, completed high school, completed vocational education, and primary school); family composition (child lives with both parents, yes vs. no); and parents' origin (2, 1, or 0 parents of Danish origin). From the *CHN records*: Concern about the mother's mental health in the first 6 months after delivery, defined as “signs of depressive mood, anxiety, sleep problems, neglect of overt problems, or referred to psychiatric care” (dichotomized into concern at 0 vs. at least one home visit); and concerns about the parent–child relationship in the first 6 months after delivery, defined as any deviation from the following description: “The child is attended to; has appropriate clothing; the parents offer the child stimulating activities, are calm and confident in their behavior resulting in a positive interaction; the parents can detect and meet the child's needs; the parents are aware of the child's weeping and can comfort the child; the parents understand and respond properly to older siblings’ reactions” (dichotomized into concern at 0 vs. at least one home visit).

### 2.3 Statistical procedures

The first step was contingency tables for the inspection of data and use of the chi-square test for heterogeneity. The second step was logistic regression analysis of the association between regulatory problems at the age of 8–10 months and diagnosed mental disorders from the age of 11 months until the eighth birthday, adjusted for the abovementioned covariates. The logistic regression analysis used RPs (one at a time, followed by a combination of sleeping and eating problems) as independent variables with diagnosed mental disorders as the outcome variables. The adjustment for covariates included three steps. Model 1 adjusts for all prenatal and perinatal conditions (sex, parity, gestational age, birth weight, congenital malformation, pregnancy complications, cesarean section, Apgar score, mother's age, father's age, parents’ education, parents' employment, family composition, parents' origin). Model 2 adjusts for exposures within the first 6 postnatal months as measured by the CHN's concern about the mother's mental health and the CHN's concerns about the parent–child relationship. Finally, Model 3 adjusts for the entire list of covariates from Models 1 and 2.

### 2.4 Data protection and ethical issues

The data collection was approved by the Research & Innovation Organization at the University of Southern Denmark (registration number 11,667) and the Danish Regional Council (registration number R-22030405), and it complied with national regulations of data protection and consent. Data were passed on from the municipalities to the National Institute of Public Health, as described in the Data Protection Legislation, and comply with the Danish codex for integrity in research (35). Linkage with register data was administered by Statistics Denmark and the involved researchers did not have access to personal identification.

## 3 Results

### 3.1 Descriptive information

In Table 1, the characteristics of the eligible and the applied study populations are illustrated by all the included variables. The composition of the eligible and the applied study populations are almost identical. The proportion of infants with sleep problems, eating problems, combined sleeping, and eating problems and problems with incessant crying was 7.7%, 19.1%, 3.6%, and 0.7%, respectively. Of the eligible study population, 4% had been diagnosed with at least one mental disorder in the period between 11 months and 8 years.

**Table 1:** The eligible and applied study populations characterized by all included variables.^a^

	Eligible total study population (*N* = 36,338) *n* (%)	Applied study populations			
		Sleeping problems subpopulation (*N* = 31,322) *n* (%)	Eating problems subpopulation (*N* = 26,437) *n* (%)	Sleeping and eating subpopulation (*N* = 25,960) *n* (%)	Incessant crying subpopulation (*N* = 13,987) *n* (%)
Regulatory problems at age 8–10 months					
Sleeping problems	—	2,403 (7.7)	—	—	—
Eating problems	—	—	5,053 (19.1)	—	—
Sleeping or eating problems	—	—	—	5,794 (22.3)	—
Sleeping and eating problems	—	—	—	942 (3.6)	—
Incessant crying problems	—	—	—		93 (0.7)
Diagnosed mental disorder					
At least one diagnosed mental disorder between 11 months and 8 years	1,439 (4.0)	1,246 (4.0)	1,064 (4.0)	1,043 (4.0)	545 (3.9)
At least one diagnosed behavioral or emotional disorder	710 (2.0)	622 (2.0)	533 (2.0)	524 (2.0)	281 (2.0)
At least one diagnosed neurodevelopmental disorder including disorders of intellectual disability, ASD, and ADHD	970 (2.7)	840 (2.7)	710 (2.7)	697 (2.7)	364 (2.6)
Autism spectrum disorders (F84)	456 (1.3)	404 (1.3)	345 (1.3)	340 (1.3)	193 (1.4)
Disorders of hyperactivity and inattention (F90, F98.8)	500 (1.4)	429 (1.4)	366 (1.4)	362 (1.4)	203 (1.5)
Intellectual disability (F70–79)	194 (0.5)	162 (0.5)	134 (0.5)	130 (0.5)	59 (0.4)
Perinatal covariates					
Male sex	18,606 (51.2)	15,933 (50.9)	13,420 (50.8)	13,127 (50.6)	7,235 (51.7)
First born child	14,646 (40.4)	12,793 (40.8)	10,797 (40.8)	10,601 (40.8)	5,753 (41.1)
Gestational age <37 weeks	2,278 (6.3)	1,9741 (6.2)	1,606 (6.1)	1,573 (6.1)	844 (6.0)
Birth weight <2,500 g	1,635 (4.5)	1,388 (4.4)	1,138 (4.3)	1,110 (4.3)	625 (4.5)
Birth weight ≥4,000 g	6,305 (17.4)	5,485 (17.5)	4,559 (17.2)	4,480 (17.3)	2,423 (17.3)
Congenital malformation	2,188 (6.0)	1,896 (6.1)	1,614 (6.1)	1,581 (6.1)	840 (6.0)
Pregnancy complications	3,050 (8.4)	2,645 (8.4)	2,279 (8.6)	2,237 (8.6)	1,098 (7.9)
Cesarean section	3,050 (21.0)	6,537 (20.9)	5,677 (21.5)	5,562 (21.4)	2,870 (20.5)
Apgar score <9	832 (2.3)	722 (2.3)	590 (2.2)	584 (2.3)	312 (2.2)
Mother's age <25 years	4,398 (12.1)	3,699 (11.8)	3,076 (11.6)	2,997 (11.5)	1,689 (12.1)
Father's age <25 years	2,293 (6.3)	1,905 (6.1)	1,571 (5.9)	1,538 (5.9)	886 (6.3)
Family covariates					
High parental level of education^b^	10,601 (29.2)	9,167 (29.3)	7,929 (30.0)	7,844 (30.2)	4,180 (29.9)
Child lives with both parents	32,191 (88.6	27,902 (89.1)	23,477 (88.8)	23,067 (88.9)	12,358 (88.4)
Two parents of Danish origin	27,417 (75.5)	23,886 (76.3)	20,166 (76.3)	19,822 (76.4)	10,468 (74.8)
Two parents in employment^c^	30,229 (83.2)	26,291 (83.9)	22,277 (84.3)	21,889 (84.3)	11,911 (85.2)
Concerns about the mother's mental health in the first 6 months after delivery	9,945 (27.4)	8,538 (27.3)	7,197 (27.2)	7,034 (27.1)	3,827 (27.4)
Concerns about parent–child relationships in the first 6 months after delivery	3,944 (10.9)	3,304 (10.6)	2,667 (10.1)	2,571 (9.9)	1,378 (9.9)

^a^ ASD, autism-spectrum disorders; ADHD, attention-deficit/hyperactivity disorder.

^b^ Parents’ educational attainment at the child's birth, categorized into five hierarchical levels from higher education (5 or more completed years at university) to primary school only (9 years at school); each child was categorized by the parent in the household with the highest education.

^c^ In employment and/or education, for mothers defined as before maternity leave; sickness benefit included in employment. Data on employment refer to the year before the year of birth.

### 3.2 Logistic regression analyses

Tables 2–5 show the odds ratio (OR) (95% confidence interval (CI)) for mental disorders between the ages of 11 months and 8 years by RPs at the age of 8–10 months. A significant association was seen between sleeping problems at the age of 8–10 months and at least one behavioral or emotional disorder (OR 1.39 (95% CI 1.07–1.81)) (Table 2). The associations between sleeping problems and mental disorders attenuated when adjusted for the covariates measured within the first 6 postnatal months (concerns about the mother's mental health and the parent–child relationship).

**Table 2:** Crude and adjusted OR (95% CI) for diagnosed mental disorders from the age of 11 months to the eighth birthday by sleeping problems at age 8–10 months (*n* = 31,322).^a^

Outcome measure	Crude OR (95% CI)	Model 1^b^	Model 2^c^	Model 3^d^
At least one diagnosed mental disorder	1.19 (0.97–1.45)	1.18 (0.97–1.45)	1.08 (0.88–1.32)	1.11 (0.90–1.36)
At least one behavioral or emotional disorder	**1.39** **(1.07–1.81)**	**1.39** **(1.07–1.81)**	1.24 (0.95–1.62)	1.28 (0.98–1.67)
At least one diagnosed neurodevelopmental disorder	1.19 (0.93–1.51)	1.18 (0.93–1.51)	1.07 (0.84–1.37)	1.11 (0.87–1.41)
Autism-spectrum disorders (F84)	1.14 (0.81–1.62)	1.16 (0.82–1.65)	1.07 (0.75–1.52)	1.11 (0.78–1.58)
Disorders of hyperactivity and inattention (F90, F98.8)	1.07 (0.76–1.52)	1.06 (0.75–1.50)	0.95 (0.67–1.35)	0.98 (0.69–1.39)
Intellectual disability (F70–79)	1.14 (0.66–1.97)	1.10 (0.63–1.91)	0.93 (0.54–1.62)	0.95 (0.54–1.65)

Bold values indicate a statistically significant result.

^a^ OR, odds ratio; CI, confidence interval.

^b^ Adjusted for sex, parity, gestational age, birth weight, congenital malformation, pregnancy complications, cesarean section, Apgar score, mother's age, father's age, parents’ education, parents’ employment, family composition, parents’ origin.

^c^ Adjusted for concerns about the mother's mental health in the first 6 months after delivery, concerns about the parent–child relationship in the first 6 months after delivery.

^d^ Adjusted for sex, parity, gestational age, birth weight, congenital malformation, pregnancy complications, cesarean section, Apgar score, mother's age, father's age, parents’ education, parents’ employment, family composition, parents’ origin, concerns about the mother's mental health in the first 6 months after delivery, concerns about the parent–child relationship in the first 6 months after delivery.

**Table 3:** Crude and adjusted OR (95% CI) for diagnosed neurodevelopmental disorders before the eighth birthday by eating problems at the age of 8–10 months (*n* = 26,437).^a^

Outcome measure	Crude OR (95% CI)	Model 1^b^	Model 2^c^	Model 3^d^
At least one diagnosed mental disorder	**1.36** **(1.18–1.57)**	**1.40** **(1.21–1.63)**	**1.25** **(1.08–1.45)**	**1.32** **(1.14–1.54)**
At least one behavioral or emotional disorder	**1.32** **(1.08–1.61)**	**1.38** **(1.12–1.70)**	1.20 (0.97–1.47)	**1.27** **(1.03–1.56)**
At least one diagnosed neurodevelopmental disorder	**1.41** **(1.19–1.68)**	**1.45** **(1.21–1.73)**	**1.28** **(1.07–1.53)**	**1.36** **(1.14–1.63)**
Autism-spectrum disorders (F84)	**1.28** **(1.00–1.65)**	**1.38** **(1.06–1.78)**	1.23 (0.95–1.59)	1.29 (0.99–1.67)
Disorders of hyperactivity and inattention (F90, F98.8)	1.17 (0.91–1.50)	1.21 (0.94–1.57)	1.03 (0.80–1.34)	1.11 (0.85–1.44)
Intellectual disability (F70–79)	**1.68** **(1.15–2.45)**	1.41 (0.95–2.10)	1.40 (0.95–2.08)	1.25 (0.84–1.85)

Bold values indicate a statistically significant result.

^a^ OR, odds ratio; CI, confidence interval.

^b^ Adjusted for sex, parity, gestational age, birth weight, congenital malformation, pregnancy complications, cesarean section, Apgar score, mother's age, father's age, parents’ education, parents’ employment, family composition, parents’ origin.

^c^ Adjusted for concerns about the mother's mental health in the first 6 months after delivery, concerns about the parent-child-relationship in the first 6 months after delivery.

^d^ Adjusted for sex, parity, gestational age, birth weight, congenital malformation, pregnancy complications, cesarean section, Apgar score, mother's age, father's age, parents’ education, parents’ employment, family composition, parents’ origin, concerns about the mother's mental health in the first 6 months after delivery, concerns about the parent–child relationship in the first 6 months after delivery.

**Table 4:** Crude and adjusted OR (95% CI) for diagnosed neurodevelopmental disorders before the eighth birthday by combined sleeping and eating problems at the age of 8–10 months (*n* = 25,960).^a^

Outcome measure	Crude OR (95% CI)	Model 1^b^	Model 2^c^	Model 3^d^
At least one diagnosed mental disorder	**1.42** **(1.06–1.89)**	**1.58** **(1.18–2.12)**	1.28 (0.96–1.71)	**1.43** **(1.07–1.92)**
At least one disorder of behavior and emotions	**1.52** **(1.03–2.23)**	**1.65** **(1.12–2.43)**	1.34 (0.91–1.98)	1.46 (0.98–2.16)
At least one diagnosed neurodevelopmental disorder	**1.55** **(1.11–2.17)**	**1.76** **(1.26–2.47)**	1.38 (0.99–1.93)	**1.60** **(1.14–2.26)**
Autism-spectrum disorders (F84)	1.58 (0.99–2.53)	**1.89** **(1.18–3.03)**	1.48 (0.93–2.37)	**1.73** **(1.07–2.79)**
Disorders of hyperactivity and inattention (F90, F98.8)	1.15 (0.68–1.94)	1.31 (0.78–2.22)	1.02 (0.60–1.72)	1.14 (0.67–1.94)
Intellectual disability (F70–79)	1.75 (0.85–3.59)	1.65 (0.80–3.41)	1.33 (0.65–2.73)	1.37 (0.66–2.88)

Bold values indicate a statistically significant result.

^a^ OR, odds ratio; CI, confidence interval.

^b^ Adjusted for sex, parity, gestational age, birth weight, congenital malformation, pregnancy complications, cesarean section, Apgar score, mother's age, father's age, parents’ education, parents’ employment, family composition, parents’ origin.

^c^ Adjusted for concerns about the mother's mental health in the first 6 months after delivery, concerns about the parent–child relationship in the first 6 months after delivery.

^d^ Adjusted for sex, parity, gestational age, birth weight, congenital malformation, pregnancy complications, cesarean section, Apgar score, mother's age, father's age, parents’ education, parents’ employment, family composition, parents’ origin, concerns about the mother's mental health in the first 6 months after delivery, concerns about the parent-child-relationship in the first 6 months after delivery.

**Table 5:** Crude and adjusted OR (95% CI) for diagnosed neuro-developmental disorders before the eighth birthday by incessant crying at age 8–10 months (*n* = 13,987).^a^

Outcome measure	Crude OR (95% CI)	Model 1^b^	Model 2^c^	Model 3^d^
At least one diagnosed mental disorder	1.71 (0.74–3.93)	1.53 (0.66–3.59)	1.49 (0.64–3.43)	1.41 (0.60–3.32)
At least one behavioral or emotional disorder	2.21 (0.81–6.06)	1.93 (0.69–5.38)	1.85 (0.67–5.12)	1.71 (0.61–4.78)
At least one diagnosed neurodevelopmental disorder	1.69 (0.62–4.63)	1.50 (0.53–4.22)	1.49 (0.54–4.09)	1.41 (0.50–3.98)
Autism-spectrum disorders (F84)	2.40 (0.75–7.66)	2.21 (0.68–7.20)	2.20 (0.69–7.03)	2.13 (0.65–6.95)
Disorders of hyperactivity and inattention (F90, F98.8)	0.74 (0.10–5.31)	0.59 (0.08–4.37)	0.63 (0.09–4.52)	0.53 (0.07–4.01)
Intellectual disability (F70–79)	^e^	^e^	^e^	^e^

^a^ OR, odds ratio; CI, confidence interval.

^b^ Adjusted for sex, parity, gestational age, birth weight, congenital malformation, pregnancy complications, cesarean section, Apgar score, mother's age, father's age, parents’ education, parents’ employment, family composition, parents’ origin.

^c^ Adjusted for concerns about the mother's mental health in the first 6 months after delivery, concerns about the parent–child relationship in the first 6 months after delivery.

^d^ Adjusted for sex, parity, gestational age, birth weight, congenital malformation, pregnancy complications, cesarean section, Apgar score, mother's age, father's age, parents’ education, parents’ employment, family composition, parents’ origin, concerns about the mother's mental health in the first 6 months after delivery, concerns about the parent–child relationship in the first 6 months after delivery.

^e^ Not calculated, too few observations.

Significant associations were seen between eating problems at the age of 8–10 months and three of the outcome variables (Table 3): at least one diagnosed mental disorder (adjusted OR 1.32 (95% CI 1.14–1.54)); at least one behavioral or emotional disorder (adjusted OR 1.27 (95% CI 1.03–1.56)); and at least one neurodevelopmental disorder (adjusted OR 1.36 (95% CI 1.14–1.63)). Once again, the association between eating problems and mental disorders attenuated when adjusted for concerns about the mother's mental health and parent–child relationship (Table 3).

Table 4 shows the longitudinal associations of a combination of sleeping and eating problems. These combined problems were associated with a significantly increased risk of at least one mental disorder (adjusted OR 1.43 (95% CI 1.07–1.92)) and at least one neurodevelopmental disorder (adjusted OR 1.60 (95% CI 1.14–2.26)), and among them, of diagnosed autism-spectrum disorders (adjusted OR 1.73 (95% CI 1.07–2.79)), but not disorders of hyperactivity and inattention. When adjusting for exposures regarding the mother's mental health and the parent–child relationship within the first 6 postnatal months, an overall pattern of attenuations of associations was seen (Table 4).

Finally, Table 5 shows that problems of incessant crying were not associated with an increased risk of diagnosed mental disorders. In these analyses, the adjustment for covariates, both those concerning prenatal, perinatal, and postnatal conditions, resulted in a considerable reduction of the OR estimates.

## 4 Discussion

### 4.1 Main findings

More than 20% of all infants have at least one CHN-reported regulatory problem, which is a prevalence estimate corresponding to prevalence estimates reported from studies using other kind of informants (1, 17). Approximately 4% of the infants in the population received at least one diagnosis of a mental disorder between the ages of 11 months and their eighth birthday, a figure that also corresponds with findings from other studies with similar sources of data (33).

CHNs’ concerns regarding sleeping problems at the age of 8–10 months were not predictive of hospital diagnosed mental disorders before the child's eighth birthday, when adjusted for potential confounders. For comparison, Winsper and Wolke (3) found an association between parent-reported sleeping problems in infancy and childhood dysregulated behavior measured by the parent's questionnaires. This is consistent with the findings in the meta-analyses by Hemmi et al. (1) and Galling et al. (6), in which most studies were based on parents’ reports at baseline as well as at the outcome measurement. Persistent sleeping problems in infancy were also associated with behavior problems, attention, and hyperactivity problems in the studies by Schmid and Wolke (17) and the study by Baumann et al. (7). Both studies were methodologically different from our study since they included a population-based sample of neonatal high-risk children and used baseline parent interviews and neurological examinations.

We found CHNs’ concern of eating problems at the age of 8–10 months associated with an increased risk of at least one behavioral or emotional disorder, and at least one diagnosed neurodevelopmental disorder. This finding is in line with other studies using observations recorded by community physicians and health nurses (5, 13) or parent interviews (17) as baseline information in infancy and measuring the outcome at the ages of 1.5, 8, or 10–12 years. The effect size was smaller in the current study, which conducted a comprehensive adjustment for confounders.

The combination of sleeping and eating problems was predictive of at least one diagnosed mental disorder and at least one diagnosed neurodevelopmental disorder, and in particular ASD, but not ADHD. In a high-risk sample, Schmid and Wolke (17) found persistent RPs predictive of a diagnosis of ADHD at school age, even when controlled for psychosocial and neurological confounders, such as gestational age, sex, maternal age at birth, socioeconomic status (SES), parent–infant relationship index, family adversity index, psychosocial stress index, and breastfeeding. Finally, we did not find any association between incessant crying at 8–10 months of age and diagnosed mental disorders in childhood. The significance of excessive or incessant crying in infancy has been explored in several high-risk as well as community-based samples, overall suggesting an increased risk of neurodevelopmental disorders (1, 3, 7, 8, 15–17, 36). The study by Lemcke (36) had many similarities with our study: it included a large, unselected Danish study population and used hospital-based diagnoses as the outcome measure. Still, in the study by Lemcke, RPs were measured by interviews with mothers when the child was aged 6 and 18 months and children were followed until the age of 11 years, which includes a period of increasing incidence of referrals to hospital around the first year of schooling. The findings from this study point to incessant crying at 6 months and sleeping problems at 18 months being associated with later neurodevelopmental disorders, although with modest effect sizes. For comparison with our study, the lack of significance of associations might reflect an observation period, not including the peak of referrals and diagnoses of ASD and ADHD, the extensive confounder control, and finally, limited power due to a relatively small number of cases (*n* = 93).

Compared to most studies exploring the association between infancy RPs and mental health outcome later in childhood as reviewed by Hemmi et al. (1) and Galling et al. (6), the associations found in the current study were weaker overall, which may be explained by the following. First, in contrast to most prior studies but one, we used mental disorders diagnosed in the hospital settings as the outcome, thus including bias of referral regarding the most common mental health problems, namely emotional and behavioral problems, which seldomly extend the threshold of referral to hospital settings in younger ages. These kinds of problems are rather treated in a community setting or settings of private practicing psychologists (33). Second, most other studies used parent-reported questionnaires that cover the more common emotional and behavioral problems. Third, in contrast to other studies of the longitudinal associations of RPs using parents as informants at both baseline and follow-up, we used CHNs as informants at baseline, and clinical disorders diagnosed in clinical settings as the outcome. This approach reduces the risk of informant bias; however, on the other hand it might introduce bias due to referrals being associated with the expressed concern of the CHNs. Finally, in comparison with prior studies, our study highlights the possibilities of extensive confounder control using the CHNs’ records and comprehensive information from Danish national registries covering all citizens in the population (35). When including data from the first 6 months of the child's life to adjust for influences regarding the mother's mental health and the parent–child relationship, we found a clear attenuation of the longitudinal associations, in line with findings from other studies (2, 25, 27). Overall, we consider the character of baseline as well as outcome information, and the extensive confounder control applied in the present study to the weakening of the statistical associations between RPs and mental disorders.

Our study explored measures of concern of RPs recorded by CHNs working within municipality healthcare, and this particular methodological approach has to be considered when discussing the associations found. We used the CHN’s overall concern about the child's sleeping, feeding and eating, and excessive crying as the index of RPs, acknowledging that this index needs further validation. Further, making use of existing service settings as the frame of baseline assessments, leave us with unmeasured confounding due to potential intervention done within the study period from measuring exposure and outcome, including initiating referrals to hospital settings. Importantly, the content of Danish community healthcare includes the possibilities of individualized support to parents of vulnerable infants and, furthermore, possibilities of referral to specialized treatment within the municipality or the health services. Unfortunately, our data do not allow for the exploration of character or the magnitude of intervention offered in municipality settings in the time span explored. Still, the potentials of identification infancy markers of psychopathology based on the CHN (20, 22, 23) has been further explored within municipality settings in Denmark, resulting in the development of standardized measures to identify and intervene toward early developmental psychopathology (5, 24, 28, 37).

### 4.2 Strengths and limitations

The strengths of the study include the longitudinal design, the large and unselected study population, the use of health professionals' systematic assessment of RPs in infancy, including clinical diagnoses of mental disorders from national population registries, and the comprehensive adjustment for confounders using data from registers and databases with a complete coverage of the population. The major limitation of our study is the validity of the CHNs' data regarding RPs, which has not been examined. Still, the data collection by CHNs follows specific guidelines to strengthen the reliability of the recordings.

### 4.3 Implications

In this study, the CHNs’ observations followed specific guidelines and are described as concerns, which indicate that something is different or troubling (30). Future studies may benefit from using available standardized assessment methods developed and validated for CHNs' early identification of regulatory problems in a community health setting (5, 28). Moreover, it is important to further explore mediating and moderating factors influencing the longitudinal associations between RPs and mental disorders. Further, previous studies have found that children with multiple RPs are especially at risk for mental disorders (1, 3, 4, 18), and further investigation of studies characterized by CHNs’ concerns regarding multiple RPs is needed. Our study focused on referred children diagnosed in a hospital setting, leaving emotional and behavioral disorders sparsely explored as these conditions rarely lead to referral to hospital settings in younger ages. It means that we are potentially excluding many children with undiagnosed mental health problems. Wishing to include emotional and behavioral mental disorders in children calls for more global psychometric measures feasible for use in population-based research, e.g., measures such as the Strengths and Difficulties Questionnaire.

From a clinical and public health point of view, the findings from the present study highlight the relevance of identifying RPs among infants to potentially break developmental trajectories leading to mental disorders. Importantly, identifying early problems should be followed by an intervention addressing the parenting of vulnerable infants, overall helping parents to meet the needs of infants with regulatory problems, to be applied in clinical as well as in municipality settings. Danish community healthcare includes the services from CHNs, who are trained to identify children who are not developing in an age-appropriate way and to communicate with parents about the developmental needs of the child (24). Still, research is needed to further explore CHNs’ potentials regarding the prevention of mental health problems in early childhood (37), and specifically, how the existing CHN surveillance program can deliver the frame for intervention to infants with regulatory problems.

## Data Availability

The data analyzed in this study is subject to the following licenses/restrictions: To get access to data underlying this article, approval from both the Danish Regional Council and the individual municipalities is necessary. Requests to access these datasets should be directed to TP, tppe@sdu.dk.
